# Germline mutations of homologous recombination genes and clinical outcomes in pancreatic cancer: a multicenter study in Taiwan

**DOI:** 10.1186/s12929-024-01008-7

**Published:** 2024-02-13

**Authors:** Siao Muk Cheng, Yung-Yeh Su, Nai-Jung Chiang, Chih-Jung Wang, Ying-Jui Chao, Chien-Jui Huang, Hui-Jen Tsai, Shang-Hung Chen, Chi-Yen Chang, Chia-Rung Tsai, Yi-Jie Li, Chia-Jui Yen, Shih-Chang Chuang, Jeffrey Shu-Ming Chang, Yan-Shen Shan, Daw-Yang Hwang, Li-Tzong Chen

**Affiliations:** 1 National Institute of Cancer Research, National Health Research Institutes, Tainan, Taiwan; 2 Institute of Clinical Medicine, College of Medicine, National Cheng Kung University, Tainan, Taiwan; 3 Deparment of Oncology, National Cheng Kung University Hospital, College of Medicine, National Cheng Kung University, Tainan, Taiwan; 4 Department of Internal Medicine, Kaohsiung Medical University Hospital and Center for Cancer Research, Kaohsiung Medical University, Kaohsiung, Taiwan; 5 Department of Oncology, Taipei Veterans General Hospital, Taipei, Taiwan; 6 School of Medicine, College of Medicine, National Yang Ming Chiao Tung University, Taipei, Taiwan; 7 Department of Surgery, National Cheng-Kung University Hospital, College of Medicine, National Cheng Kung University, Tainan, Taiwan; 8 Department of Internal Medicine, National Cheng Kung University Hospital, College of Medicine, National Cheng Kung University, Tainan, Taiwan; 9 Division of General and Digestive Surgery, Department of Surgery, Kaohsiung Medical University Hospital, Kaohsiung Medical University, Kaohsiung, Taiwan; 10 Department of Surgery, Faculty of Medicine, Kaohsiung Medical University, Kaohsiung, Taiwan; 11 Division of Nephrology, Department of Internal Medicine, Kaohsiung Medical University Hospital, Kaohsiung Medical University, Kaohsiung, Taiwan; 12 Center for Biomarkers and Biotech Drugs, Department of Biomedical Science and Environmental Biology, Kaohsiung Medical University, Kaohsiung, Taiwan; 13 Precision Medicine Ph.D. Program, National Tsing Hua University, Hsinchu, Taiwan

**Keywords:** Next-generation sequencing, Homologous recombination, Platinum, Chemotherapy

## Abstract

**Background:**

Cancer susceptibility germline mutations are associated with pancreatic ductal adenocarcinoma (PDAC). However, the hereditary status of PDAC and its impact on survival is largely unknown in the Asian population.

**Methods:**

Exome sequencing was performed on 527 blood samples from PDAC individuals and analyzed for mutations in 80 oncogenic genes. Pathogenic and likely pathogenic (P/LP) germline variants were diagnosed according to the ACMG variant classification categories. The association between germline homologous recombination gene mutations (*gHR*^*mut*^, including *BAP1*, *BRCA1*, *BRCA2*, *PALB2*, *ATM*, *BLM*, *BRIP1*, *CHEK2*, *NBN*, *MUTYH*, *FANCA* and *FANCC*) and the treatment outcomes was explored in patients with stage III/IV diseases treated with first-line (1L) platinum-based versus platinum-free chemotherapy.

**Results:**

Overall, 104 of 527 (19.7%) patients carried germline P/LP variants. The most common mutated genes were *BRCA2* (3.60%), followed by *ATR* (2.66%) and *ATM* (1.9%). After a median follow-up duration of 38.3-months (95% confidence interval, 95% CI 35.0–43.7), the median overall survival (OS) was not significantly different among patients with *gHR*^*mut*^, non-*HR* germline mutations, or no mutation (*P* = 0.43). Among the 320 patients with stage III/IV disease who received 1L combination chemotherapy, 32 (10%) had *gHR*^*mut*^. Of them, patients receiving 1L platinum-based chemotherapy exhibited a significantly longer median OS compared to those with platinum-free chemotherapy, 26.1 months (95% CI 12.7–33.7) versus 9.6 months (95% CI 5.9–17.6), *P* = 0.001. However, the median OS of patients without *gHR*^*mut*^ was 14.5 months (95% CI 13.2–16.9) and 12.6 months (95% CI 10.8–14.7) for patients receiving 1L platinum-based and platinum-free chemotherapy, respectively (*P* = 0.22). These results were consistent after adjusting for potential confounding factors including age, tumor stage, performance status, and baseline CA 19.9 in the multivariate Cox regression analysis.

**Conclusions:**

Our study showed that nearly 20% of Taiwanese PDAC patients carried germline P/LP variants. The longer survival observed in *gHR*^*mut*^ patients treated with 1L platinum-based chemotherapy highlights the importance of germline testing for all patients with advanced PDAC at diagnosis.

**Supplementary Information:**

The online version contains supplementary material available at 10.1186/s12929-024-01008-7.

## Background

Pancreatic ductal adenocarcinoma (PDAC) is an aggressive disease with an increasing incidence and mortality globally. To date, PDAC is the third leading cause of cancer mortality in the United States with a five-year survival rate of less than 12% [1]. The poor prognosis of PDAC is mainly due to delayed diagnosis, a lack of feasible detection markers, low resectability, a high recurrence rate, high resistance to chemotherapy and radiotherapy, and limited available effective therapeutic agents [2]. The mortality of major cancer types including lung cancer, breast cancer and colorectal cancer, is steadily decreasing after the introduction of novel target therapy and immunotherapy [3]. Immune checkpoint inhibitors have demonstrated promising efficacy in a variety of cancer types, but it adds limited clinical benefits when combining with standard chemotherapy in PDAC [4]. Currently, there are no effective target therapies in PDAC except poly (ADP-ribose) polymerase (PARP) inhibitors in a small subset of metastatic PDAC patients with germline *BRCA1* or *BRCA2* mutations [5]. As a result, PDAC is predicted to become the second leading cause of cancer death in the United States by 2026 and in Germany by 2030 [3, 6]. A screening program for early diagnosis, new treatment strategies, and the discovery of target-specific drugs to decrease deaths from PDAC are urgently needed.

Given that most PDAC patients are diagnosed at advanced stage, screening programs for all comers may not cost-effective due to the relatively low incidence of PDAC. Identification of high-risk individuals is a more reasonable approach for screening to detect early stage PDAC and yield better treatment outcomes. Individuals with a family history of cancer were shown to have a greatly elevated risk of developing cancer, especially so-called *BRCA*-associated hereditary cancers, including breast, ovary, prostate, and pancreatic cancer [7–9]. Notably, approximately 5–10% of PDAC patients in the United States and Japan have a family history of the disease [10, 11]. The alterations in cancer susceptible genes such as *BRCA1*, *BRCA2*, *PALB2*, *STK11*, *PRSS1,* and *ATM* have been linked to increased lifetime risks of PDAC [12–14]. Family members of PDAC patients with germline P/LP mutations in hereditary cancer susceptible genes are the candidates for active screening programs, such as Pancreatic Cancer Early Detection (PRECEDE) Consortium [15].

The development of a new drug usually requires enormous amounts of time and money. Through the concept of precision medicine, using the right drug for the right patient at the right time, the outcomes of PDAC can be more efficiently improved, as demonstrated in the Know Your Tumor program [16]. Germline mutations of *BRCA1*, *BRCA2,* or *PALB2* were associated with improved responses to and survival under platinum-based chemotherapy [17, 18]. There is increasing evidence that PDAC patients with mutations in other homologous recombination (HR) genes can also benefit from platinum-based chemotherapy [19, 20]. Identifying patients with germline mutations in cancer susceptibility genes, especially HR genes, not only assist in screening high-risk familiar members but also benefit proper treatment selection.

Current genomic studies have investigated the germline mutation landscape of PDAC patients based on whole genome sequencing and exome sequencing. However, the hereditary mutation genes in the Asian population are poorly described. In this study, we performed exome sequencing on 527 Taiwanese PDAC blood samples to identify possible germline mutations in cancer susceptibility genes and to explore the association between germline HR genes mutations and outcomes of advanced PDAC receiving first-line (1L) platinum-based versus platinum-free chemotherapy.

## Materials and methods

### Sample acquisition and questionnaires

A multicenter prospective observation cohort that recruited PDAC patients from the National Cheng Kung University Hospital and the Kaohsiung Medical University Hospital was established in 2013 [21]. Patient recruitment is still ongoing. The current analysis included 527 patients who consented between 2013 and 2021. The review process of clinical information was retrospectively registered on January 26, 2023 (ClinicalTrials.gov Identifier: NCT05700188). Patients with multiple cancer types were excluded from this study. All patients provided written informed consent, donated 20 ml blood samples, and answered a detailed questionnaire about the age at diagnosis of any cancer and the types of cancer diagnosed in their first- and second-degree relatives. Questionnaires were completed by patients at home and sent back to the physicians.

### DNA extraction, exome sequencing, and data processing

Genomic DNA was extracted from peripheral blood samples using a Wizard® Genomic DNA Purification Kit (Promega). All samples were processed using two different WES enrichment platforms: Nextera Flex for Enrichment, an Exome panel (Illumina), and an Accel-NGSR 2S Hyb DNA Library Kit (Swift). In total, 50–1000 ng of DNA was sheared into short fragments using enzymatically (Illumina) and Covaris M220 Focused-ultrasonicator (Swift). Size selection, sequence capturing, enrichment, and elution were performed according to the manufacturer’s instructions. The size distribution of DNA libraries was then measured using 4150 TapeStation (Agilent). Finally, the validated DNA libraries were sequenced on an Illumina sequencing platform (NovaSeq 6000) with 150 bp paired-end reads. The read pair data (fastq) of each sample were generated from the sequencing system. The resulting reads were then aligned to the reference human genome sequence (GRCh37/hg19), and nucleotide variant calling was performed using the DRAGEN Enrichment app (ver 3.6.3) in the Illumina basespace sequence hub. The VCF files from the DRAGEN Enrichment app were annotated and analyzed in the CLC Genomics Workbench (ver 20) software with default parameters.

### Data analysis and variant characterization

Eighty oncogenic candidate genes associated with germline mutations were chosen for this study based on a literature review and COSMIC cancer gene census. Selected genes were divided into four nonoverlapping categories according to previous reports. Mutations in 12 genes, including *BAPI*, *BRCA1*, *BRCA2*, *PALB2*, *ATM*, *BLM*, *BRIP1*, *CHEK2*, *NBN*, *MUTYH*, *FANCA*, and *FANCC*, were considered as homologous recombination (HR) genes (Additional file 1: Table S1) [22–33]. Variants were retained if they passed the quality criteria (variant count of ≥ 2 and variant allele frequency between 20 and 80%). Pathogenic probabilities according to The American College of Medical Genetics and Genomics (ACMG) variant classification categories were determined by the VarSome (ver.11.4) prediction database. Only pathogenic, likely pathogenic, and variants of uncertain significance (VUS) were analyzed in our study. Pathogenic and likely pathogenic variants were also filtered if the minor allele frequency (MAF) was > 1% in the 1000 Genomes database, Genome Aggregation Database (gnomAD), and dbSNP database. Annotated variants were further assessed in common databases such as ClinVar and HGMD.

### Statistical analysis

Fisher’s exact test was performed to determine the cancer types among those with family cancer history between carriers and non-carriers of pathogenic variants. A paired t-test was applied to compare the ages of patients with or without family history of BRCA-associated hereditary cancers, with or without germline mutation and with or without *gHR*^*mut*^. Overall survival was calculated from diagnosis to death due to any cause or censored in the case of loss to follow-up or data cut-off. Two-sided *P* < 0.05 was considered statistically significant.

## Results

### Population characteristics

The clinical features of 527 PDAC patients are summarized in Table 1. In total, 298 males (56.5%) and 229 females (43.5%) participated in this study. The median age of diagnosis of the entire cohort was 62.5 years, ranging from 27.0 to 93.9 years. A total of 48 patients (9.1%) had stage I disease, 111 (21.1%) had stage II disease, 120 (22.8%) had stage III disease, and 248 (47.1%) had stage IV disease upon diagnosis. The most common primary location of the tumor was in the head (37.0%), followed by the body (20.5%), tail (17.8%), and uncinate process (3%). The remaining 114 (21.6%) cases had tumors located in overlapping areas. Family history of any cancer, which included first- and second-degree relatives, was observed in 54.3% of patients.

**Table 1:** Clinical and demographic characteristics of study participants

	*N*	Percent (%)
Number of patients	527	
Gender		
Male	298	56.5
Female	229	43.5
Age		
Median (range)	62.5 (27.0–93.9)	
Primary tumor location		
Head	195	37.0
Uncinate	16	3.0
Body	108	20.5
Tail	94	17.8
Overlapping area	114	21.6
Clinical stage		
I	48	9.1
II	111	21.1
III	120	22.8
IV	248	47.1
Family cancer history		
Yes	286	54.3
No	236	44.8
Unknown	5	0.9

### Germline mutational landscape of pancreatic cancer

**Fig. 1:**
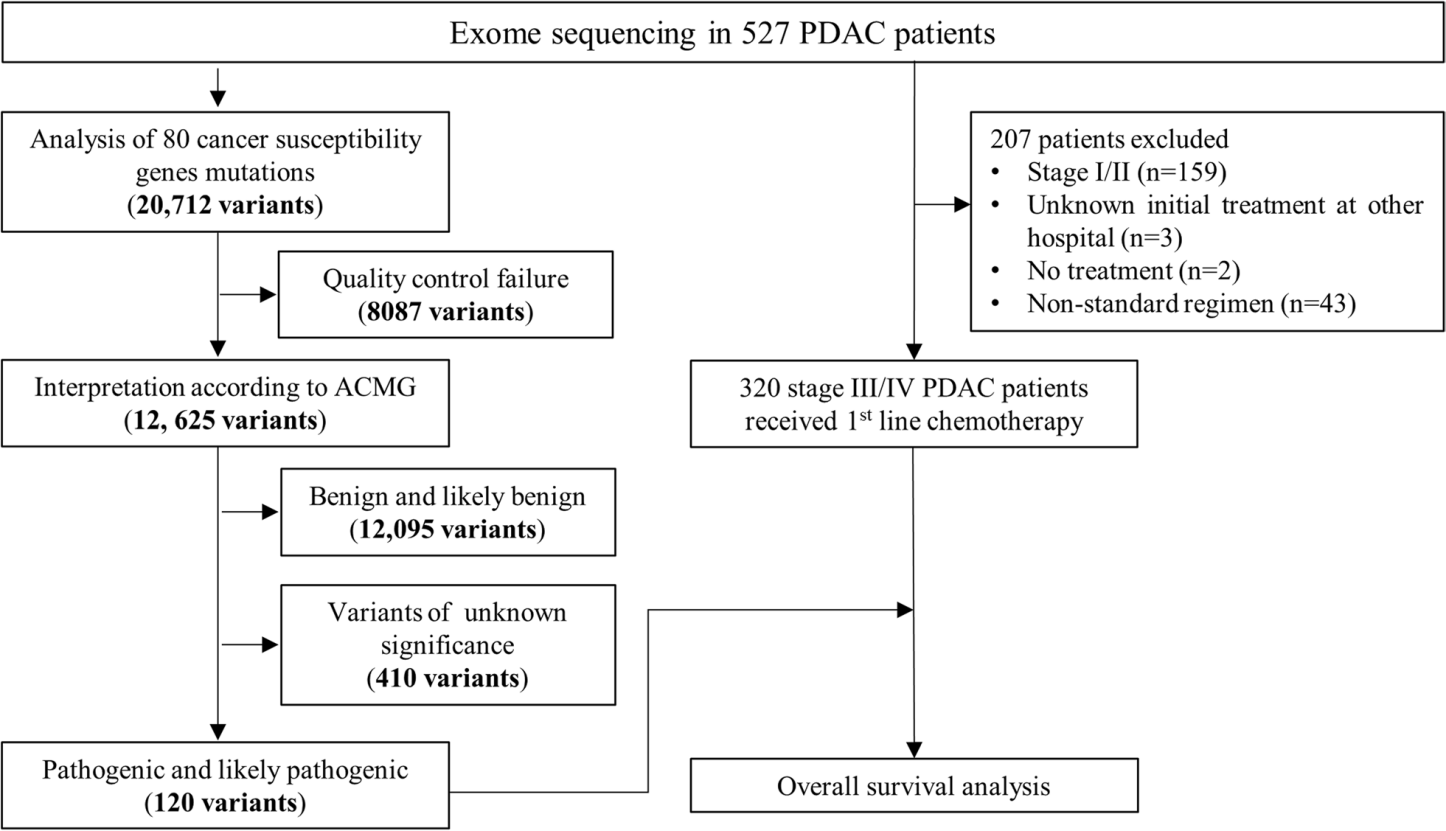
Analysis flowchart of mutation variants in 527 patients

**Fig. 2:**
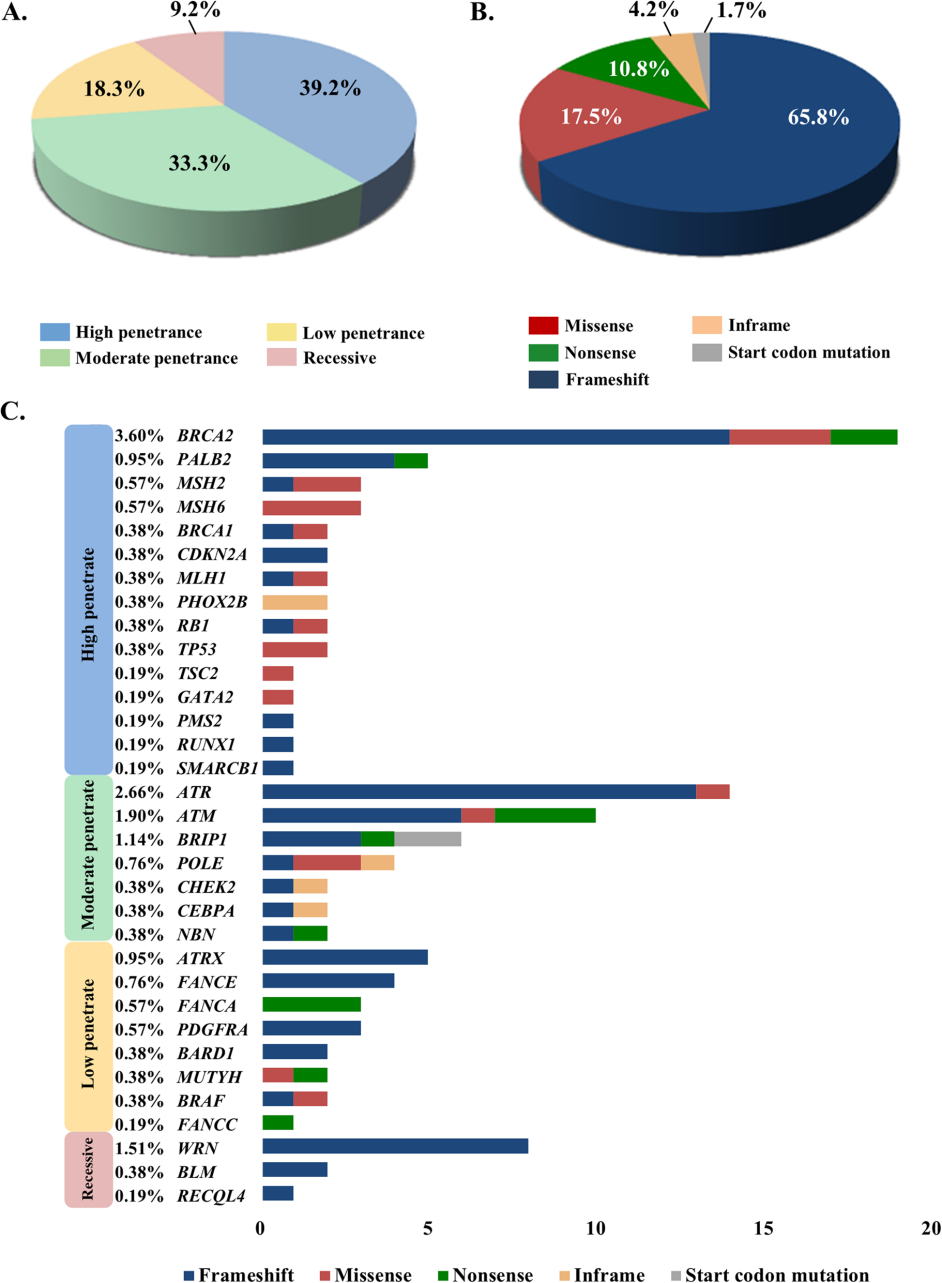
The distribution of 120 pathogenic and likely pathogenic (P/LP) germline mutations in PDAC patients. **A** The frequency of P/LP mutation genes in four categories (low-, moderate-, high- and recessive penetrance). **B** The frequency of the mutational type of P/LP mutation genes. **C** The landscape of P/LP mutation genes in patients. The distribution of mutational type was included in the figure

To investigate the susceptibility of germline mutational genes in pancreatic cancer, we performed exome sequencing of all PDAC patients in the study cohort. The mean coverage in the exome sequencing data was 62 (range, 19–164). In total, 80 genes were selected because of the potential effects of germline mutations, and the predicted pathogenicity of variants was determined according to ACMG variant classification categories. As shown in Fig. 1, a total of 20,712 germline variants were identified, and 12,625 variants passed the quality control. Of these, 62 (0.49%) were considered pathogenic variants, 58 (0.46%) were likely pathogenic variants, 410 (3.25%) were variants of uncertain significance, 744 (5.89%) were likely benign, and 11,351 (89.91%) were benign variants. Among 527 PDAC patients, a total number of 104 (19.7%) patients carried 120 P/LP variants, 15 patients had two or more P/LP variants. In our classification categories, 47 (39.2%) P/LP variants were considered to have high penetrance, followed by 40 (33.3%) with moderate penetrance, 22 (18.3%) with low penetrance, and 11 (9.2%) indicating a recessive condition (Fig. 2A). A total number of 120 P/LP variants included 21 (17.5%) missense mutations, 13 (10.8%) nonsense mutations, 79 (65.8%) frameshift mutations, 5 (4.2%) inframe mutations, and 2 (1.7%) start codon mutations (Fig. 2B). The most common mutated genes were *BRCA2* (3.6%), followed by *ATR* (2.66%) and *ATM* (1.9%) (Fig. 2C). Here, we summarize the most frequent P/LP variants (case ≥ 3) in Table 2, which includes *ATM* (p.Lys468fs), *ATR* (p.Ile774fs), and *WRN* (p.Lys168fs). Detailed characteristics of the 120 pathogenic and likely pathogenic mutations carried in 104 patients were summarized in Additional file 1: Table S2.

**Table 2:** The most frequently pathogenic and likely pathogenic germline variants (n > 3) in cancer susceptibility genes

Gene	Refseq transcripts	HGVS coding	HGVS protein	ACMG	Degrees	dbSNP	MAF	ClinVar	Patient ID
*ATM*	NM_000051.4	c.1402_1403del	p.Lys468fs	Pathogenic	PVS1: very strong, PP5: very strong, PM2: supporting	rs587781347	0.000019	Pathogenic	H0432, H11204, H4414, KH0065
*ATR*	NM_001184.4	c.2320dup	p.Ile774fs	Pathogenic	PVS1: very strong, PP5: strong	rs757500301	0.00018	Pathogenic	H10846, H3731, H0028, H0626, H2933, H4039, H5930, H6833
*WRN*	NM_000553.6	c.502_503del	p.Lys168fs	Pathogenic	PVS1: very strong, PP5: moderate, PM2: supporting	rs776785728	0.000064	Pathogenic	H0083, H0410, H11216, H11993

*MAF* minor allele frequency, *ACMG* American College of Genetics and Genomics, *PVS* very strong pathogenicity, *PP* supporting pathogenicity, *PM* moderate pathogenicity, *PS* pathogenic strong

### Associations between family cancer history and age distribution in PDAC patients

**Fig. 3:**
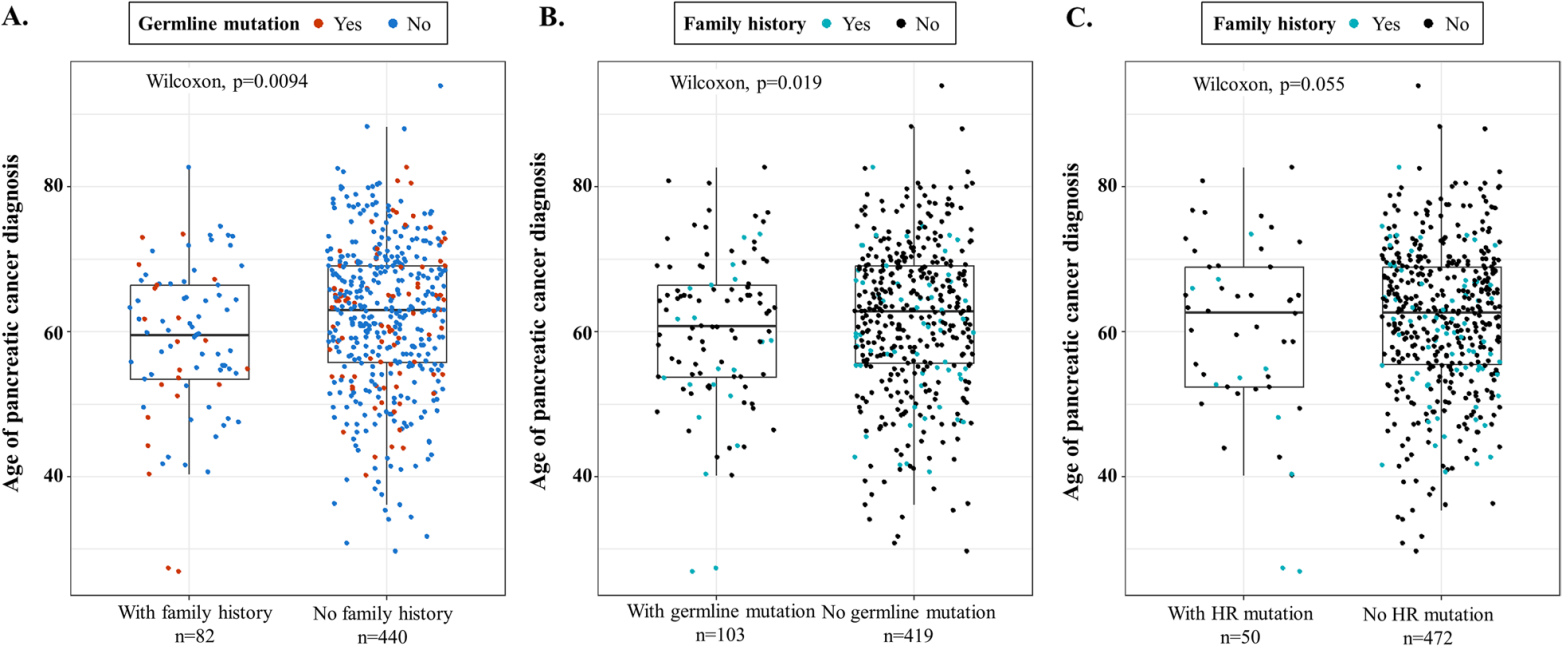
Distribution of pancreatic cancer diagnosed age. **A** Patients with or without family history of breast, ovary, prostate, and pancreatic cancer. **B** Patients with or without germline mutation in 80 oncogenic candidate genes. **C** Patients with or without germline mutation in 12 homologous recombination genes

Overall, 286 of 527 (54.3%) patients had a family cancer history in our study (Table 1) and the most common types of cancer among the families were liver cancer (13.7%), lung cancer (9.1%), pancreatic cancer (8.4%), colon cancer (7.8%), and breast cancer (5.9%). A family history of pancreatic cancer was identified in 8.7% (9/104) of P/LP variants carriers and 8.3% (35/423) of non-carriers. Detailed family history information was shown in Additional file 1: Table S3. Excluding five patients with unknown family history, 82 of 522 (15.7%) patients had a family history of *BRCA*-associated hereditary cancers including breast, ovary, prostate, and pancreatic cancer. The diagnosed ages of patients with a family history of these four cancer types were significantly younger than the ages of those without a family history (median age 59.6 vs. 63.0 years old, *P* = 0.0094) (Fig. 3A). Conversely, the diagnosed ages of patients with germline mutations in the 80 oncogenic candidate genes or *gHR*^*mut*^ were not significantly different than those of their counterparts (Fig. 3B, C).

### Impact of germline HR gene alterations on chemotherapy efficacy

**Fig. 4:**
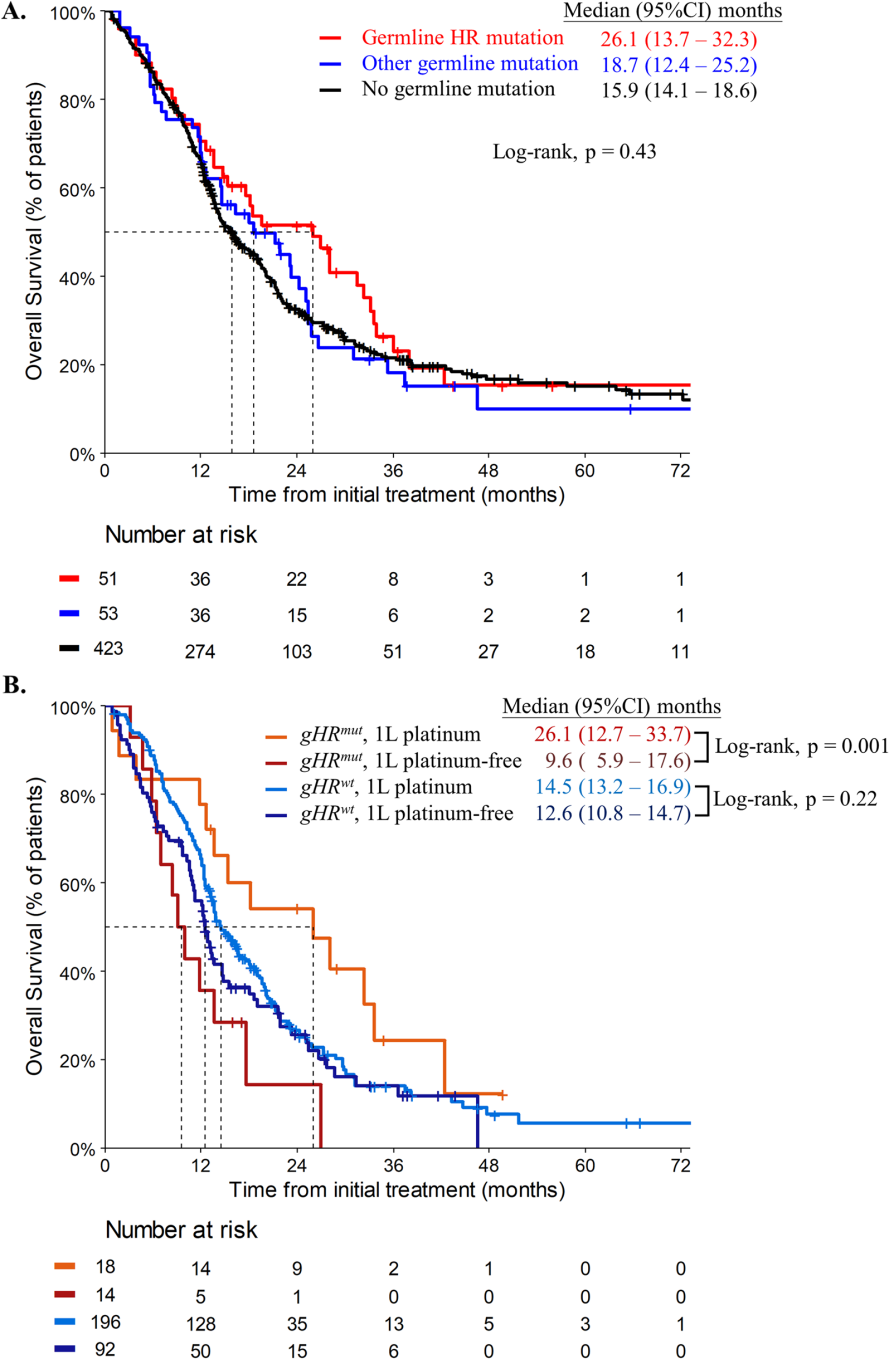
Outcome of PDAC patient in homologous recombination (HR) gene mutations status. **A** Median overall survival of 527 PDAC patients with germline HR gene mutations, other gene mutations or no mutation. **B** Median overall survival of 320 stage III/IV PDAC patients with or without HR gene mutations treated with 1L platinum-based or platinum-free chemotherapy

As of December 31, 2021, the median duration of follow-up was 38.3 months (95% confidence interval [95% CI] 35.0–43.7). P/LP variant carriers tend to have a better median OS than non-carriers in both stage I/II (33.2 months vs. 25.1 months, *P* = 0.9) and stage III/IV (18.0 months vs. 13.2 months, *P* = 0.062) cancer (Additional file 1: Fig. S1). Patients with *gHR*^*mut*^ exhibited a numerically better median OS of 26.1 months (95% CI 13.7–32.3), compared to 18.7 months (95% CI 12.4–25.2) in those with non-HR germline mutations and 15.9 months (95% CI 14.1–18.6) in patients without germline mutations, *P* = 0.43 (Fig. 4A).

To further investigate the correlation between germline mutations and chemotherapy efficacy, patients who received first-line chemotherapy were identified for detailed analysis. A total of 159 patients with stage I/II PDAC were excluded, as chemotherapy is not the standard of care at this stage. Among the 368 patients with stage III/IV diseases, 3 patients who received unknown treatments at other hospital, 2 untreated patients, and 43 fragile patients who underwent non-standard including gemcitabine monotherapy were excluded. Finally, 320 patients with stage III/IV diseases underwent first-line combination chemotherapy, including 32 patients (10%) with *gHR*^*mut*^ and 288 patients with germline HR wild-type (*gHR*^*wt*^), were included in the analysis (Figs. 1 and 4B). The baseline characteristics, including sex, age, tumor location, stage, family history, ECOG, albumin, CA-19.9 and 1L chemotherapy regimens, platinum-based or platinum-free chemotherapy were not significantly different between patients with or without *gHR*^*mut*^ (Table 3). Overall, 214 patients were treated with 1L platinum-based chemotherapy, SLOG (S1, leucovorin, oxaliplatin and gemcitabine) in 153 (47.8%), modified FOLFIRINOX in 34 (10.6%) and GOFL (gemcitabine, oxaliplatin, fluorouracil, and leucovorin) in 27 (8.4%). A total of 106 patients received 1L platinum-free chemotherapy, including gemcitabine plus nab-paclitaxel in 54 (16.9%) and gemcitabine plus S1 in 52 (16.3%) (Table 3).

**Table 3:** Characteristics of 320 patients who received first-line platinum-based or platinum-free chemotherapies with different HR gene alteration statuses

	*gHR*^*mut*^ (N = 32)	*gHR*^*wt*^ (N = 288)	Overall (N = 320)
Gender			
Female	16 (50.0%)	125 (43.4%)	141 (44.1%)
Male	16 (50.0%)	163 (56.6%)	179 (55.9%)
Age, median (IQR)	61.7 (52.5, 68.9)	62.8 (55.3, 68.6)	62.8 (55.2, 68.8)
Location			
Head	10 (31.3%)	74 (25.7%)	84 (26.3%)
Uncinate process	0 (0%)	12 (4.2%)	12 (3.8%)
Body	6 (18.8%)	74 (25.7%)	80 (25.0%)
Tail	6 (18.8%)	59 (20.5%)	65 (20.3%)
Overlapping area	10 (31.3%)	69 (24.0%)	79 (24.7%)
Stage			
III	13 (40.6%)	94 (32.6%)	107 (33.4%)
IV	19 (59.4%)	194 (67.4%)	213 (66.6%)
Family history			
Yes	19 (59.4%)	160 (55.6%)	179 (55.9%)
No	12 (37.5%)	125 (43.4%)	137 (42.8%)
Unknown	1 (3.1%)	3 (1.0%)	4 (1.3%)
ECOG PS			
0–1	28 (87.5%)	260 (90.3%)	288 (90.0%)
≥ 2	4 (12.5%)	28 (9.7%)	32 (10.0%)
Baseline albumin, g/dL			
Median (IQR)	4.200 (3.700, 4.500)	4.100 (3.800, 4.400)	4.100 (3.800, 4.400)
Not recorded	7 (21.9%)	85 (29.5%)	92 (28.8%)
Baseline CA-19.9, U/mL			
Median (IQR)	2244 (255.1, 5772)	444.5 (64.6, 3030)	471.0 (76.0, 3561)
Not recorded	5 (15.6%)	50 (17.4%)	55 (17.2%)
Enrollment in clinical trial			
Yes	27 (84.4%)	220 (76.4%)	247 (77.2%)
No	5 (15.6%)	68 (23.6%)	73 (22.8%)
1L platinum-based			
SLOG	12 (37.5%)	141 (49.0%)	153 (47.8%)
mFOLFIRINOX	1 (3.1%)	33 (11.5%)	34 (10.6%)
GOFL	5 (15.6%)	22 (7.6%)	27 (8.4%)
1L platinum-free			
Gem + nab-paclitaxel	7 (21.9%)	47 (16.3%)	54 (16.9%)
Gem + S1	7 (21.9%)	45 (15.6%)	52 (16.3%)

The median OS of patients with *gHR*^*mut*^ receiving 1L platinum-based chemotherapy was significantly better than those receiving platinum-free chemotherapy, 26.1 months (95% CI 12.7—33.7) versus 9.6 months (95% CI 5.9–17.6) (*P* = 0.001, Fig. 4B). However, the median OS was not significantly different between *gHR*^*wt*^ patients treated with 1L platinum-based and platinum-free chemotherapy, with a median OS of 14.5 months (95% CI 13.2–16.9) and 12.6 months (95% CI 10.8–14.7), respectively (*P* = 0.22, Fig. 4B). All baseline characteristics were not significantly different for those with *gHR*^*mut*^ receiving 1L platinum-based or platinum-free chemotherapy (Additional file 1: Table S4).

Overall, 37 of 107 (34.6%) patients with stage III disease underwent conversion surgery after first-line chemotherapy, including 6 of 13 patients (46.2%) in the *gHR*^*mut*^ group and 31 of 94 patients (33.0%) in the *gHR*^*wt*^ group (Fisher’s exact test, *P* = 0.37). The median OS of patients with and without conversion surgery was 33.7 months (95% CI 29.7–NE) and 16.5 months (95% CI 13.7–20.2), respectively. (Additional file 1: Fig. S2).

### Adjustment of confounding factors

**Fig. 5:**
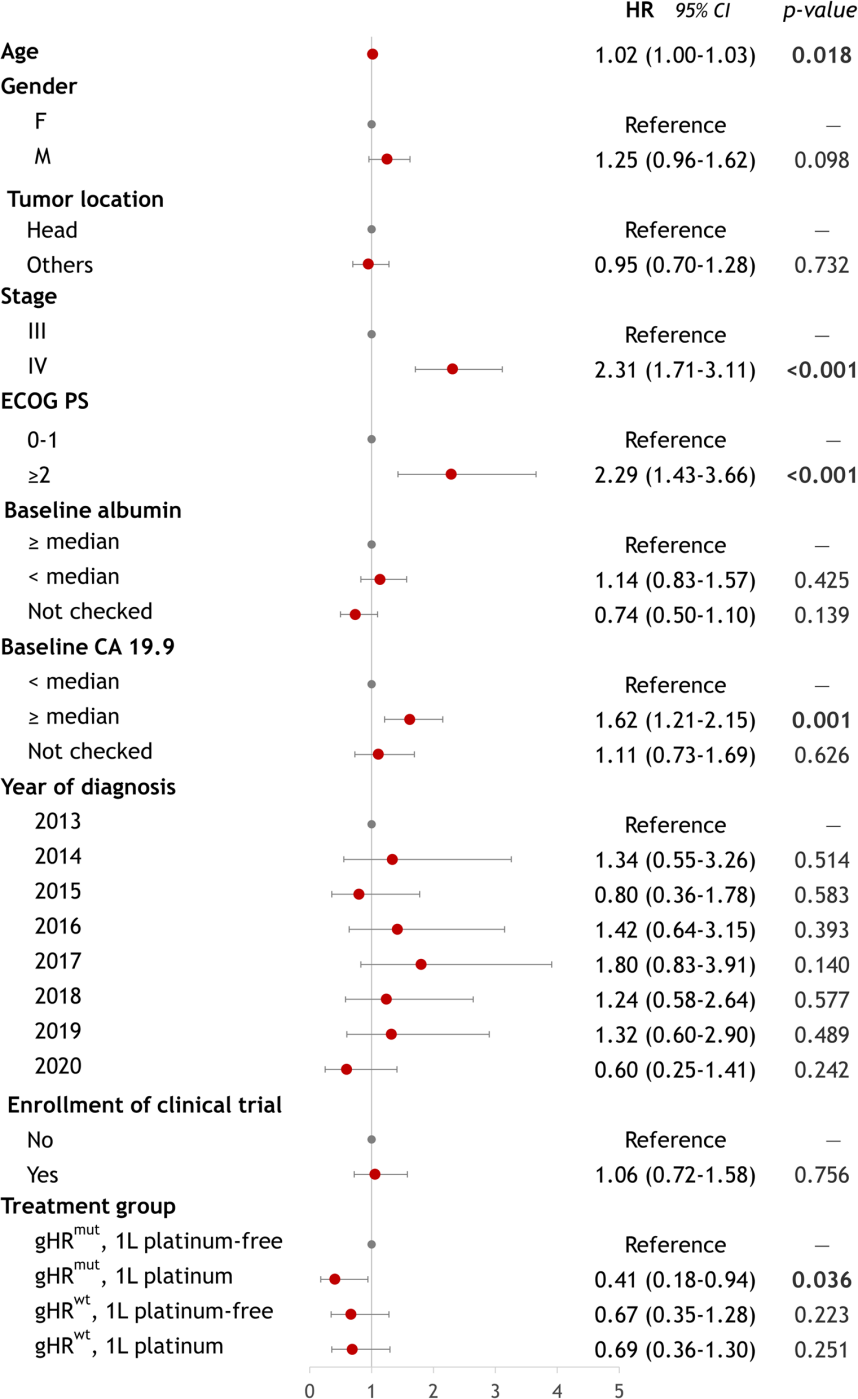
Multivariable Cox regression analysis. Grey dots represent the reference group, while the red dot indicates the hazard ratio (HR) with a depicted 95% confidence interval

Potential confounding factors were adjusted using a multivariable Cox regression analysis, which included age, gender, tumor location, stage, ECOG PS, baseline albumin, baseline CA 19.9, year of diagnosis, and enrollment in the clinical trial. After this adjustment, age, ECOG PS, and baseline CA 19.9 were identified as significant prognostic factors. Additionally, patients with *gHR*^*mut*^ treated with 1L platinum-based chemotherapy continued to show a significant improvement in survival compared to those treated with platinum-free chemotherapy, with a hazard ratio of 0.41 (95% CI 0.18–0.94, *P* = 0.036) (Fig. 5).

Due to the delayed reimbursement of nab-paclitaxel (reimbursed in 2019) and FOLFIRINOX (reimbursed in 2021), a great proportion of patients in the current study participated in investigator-initiated trials (IITs), as the treatment options were very limited in Taiwan at that time (Additional file 1: Fig. S3) [34–42]. Therefore, during the study period (2013–2020), the majority of patients received SLOG, either in IITs or in daily practice, while nab-paclitaxel plus gemcitabine was used after its reimbursement in 2019 (Additional file 1: Fig. S4). Overall, 73 of the 320 patients with advanced PDAC were enrolled in the IITs. There were no significant differences in patient survival between those with *gHR*^*mut*^ and those with *gHR*^*wt*^, regardless of whether they were enrolled in the clinical trial (*P* = 0.94) (Additional file 1: Fig. S5). Enrollment in the clinical trial was not prognostic significance in the multivariate Cox regression analysis (hazard ratio 1.06, 95% CI 0.72–1.58, *P* = 0.756) (Fig. 5).

For the sensitivity analysis, five patients were excluded due to unknown initial treatments at referral hospital in three and no treatment in two. The remaining 363 patients with advanced stage cancer were analyzed. The regimen is listed in Additional file 1: Table S5. Patients who received gemcitabine monotherapy had extremely poor outcomes, with a median OS of 5.1 months compared to those who received other regimens, whose median OS ranged from 12.0 to 15.7 months (Additional file 1: Fig. S6A). The survival benefits of 1L platinum-based chemotherapy in patients with *gHR*^*mut*^ remained significant when all 363 stage III/IV patients were included in the analysis (Additional file 1: Fig. S6B).

## Discussion

In this study, DNA from PBMC of 527 patients with PDAC were sequenced via exome sequencing, and the characteristics of 80 oncogenic candidate genes were analyzed. To our knowledge, our study is, to date, the largest PDAC cohort with comprehensive germline genetic profiling and treatment outcomes in the Asian population and may serve as a useful resource for investigating the mechanisms underlying PDAC. Overall, we identified 120 P/LP variants in 104 (19.7%) PDAC patients. Furthermore, more than half of them were high-penetrance genes. Since the prognosis of PDAC is extremely poor, and early diagnosis provides the best chance of cure nowadays, genetic counseling and cancer screening programs should be recommended to at-risk families [43].

The median age of PDAC diagnosis in our study was significantly younger in patients with a family history of *BRCA*-associated hereditary cancers, including breast, ovarian, prostate, and pancreatic cancer. However, there was no significant difference in the age of diagnosis between patients with or without germline mutations in the 80 oncogenic candidate genes or the 12 HR genes, suggesting that other genes, and/or environmental and lifestyle factors may also play an important role in tumorigenesis. Future studies should consider analyzing not only these 80 oncogenic candidate genes but also additional genes. However, germline variants in other genes may be rare, making it challenging to assess their impacts in a cohort of this size. A larger-scale nationwide or international collaboration program, such as the PRECEDE Consortium [44], is needed to address this issue.

In our study, 54 of 120 (45%) P/LP variants were of HR genes. The median OS was not significantly different among patients with *gHR*^*mut*^, other germline mutations or no mutations (*P* = 0.43, Fig. 4A), suggesting that *gHR*^*mut*^ per se may not be a prognostic factor. On the other hand, patients with *gHR*^*mut*^ who received 1L platinum-based chemotherapy had significantly better OS compared to those who received platinum-free chemotherapy. However, the median OS was not significantly different between patients with *gHR*^*wt*^ who received 1L platinum-based versus platinum-free chemotherapy. Our study confirmed previous findings that PDAC patients with *gHR*^*mut*^ have better responses and superior overall survival when treated with platinum-based chemotherapy [32, 45–47]. These findings suggest that *gHR*^*mut*^ may serve as a predictive biomarker for platinum-based chemotherapy.

Germline *BRCA1/2* mutations have been linked to inherited risks of PDAC. In total, 3.98% of patients harbored germline P/LP variants in either *BRCA1* or *BRCA2* in our cohort, which is similar to the results of investigations among Western (3.5%) and Japanese (3.4%) populations [13, 48]. The PARP inhibitor olaparib is approved by the U.S. Food and Drug Administration as maintenance therapy only in metastatic PDAC patients with germline *BRCA1/2* mutations [5]. While germline *BRCA1/2* mutation consisted of only 2–3% of PDAC patients, the broad definition of *gHR*^*mut*^ was identified in more than 10% of PDAC patients. A previous retrospective study suggested that olaparib is also effective in patients with *gHR*^*mut*^ other than *BRCA1/2* [49]. Germline genetic profiling of all advanced PDAC patients will help to identify candidates who might benefit from PARP inhibitors. The overall incidence of *gHR*^*mut*^ in our cohort was 9.7% (51/527), which is in line with the results of previous reports (9.7–16.5%) [32, 50–52]. The incidence of *gHR*^*mut*^ in current study might be underestimated because patients with multiple primary cancers, who were more likely to harbor germline cancer susceptibility gene mutations, were excluded from initial study [53].

*ATM* is involved in DNA double-strand break repair and is necessary to maintain genome stability. *ATM* mutations cause ataxia-telangiectasia (AT) and have been associated with a risk of breast cancer [54, 55]. Yang et al*.* suggested that the primary types of *ATM* mutations are nonsense and frameshift mutations and that mutation carriers among Chinese *BRCA1/2*-negative breast cancer patients are more likely to have a family history of cancer [56]. In our results, ten patients (1.90%, 10/527) harbored P/LP mutations in the *ATM* gene, and five (50%, 5/10) carriers had a family history of cancer. *ATM* p.Lys468fs (40%, 4/10) was the most prevalent *ATM* mutation in our study, which was observed in AT patients and may cause the development of AML in children [57]. However, the effect of this frameshift mutation on the risk of pancreatic cancer remains undetermined.

Before 2019, only gemcitabine and S1 were reimbursed for advanced PDAC treatment in Taiwan. Given the limited treatment options available at that time, one of the primary objectives of our IITs was to provide relatively safe and effective treatment for them. Consequently, the inclusion criteria in the trial closely resembled those of our standard approach in daily patient care, which was reflected by the lack of a significant difference in survival between patients enrolled and not enrolled in the clinical trial. Both the GOFL and SLOG regimens were incorporated into daily practice in our hospitals prior to the reimbursement of nab-paclitaxel and FOLFORINOX [58–62]. Patients treated with gemcitabine monotherapy (n = 21), usually prescribed for fragile patients as recommended in the majority of treatment guidelines, were excluded in the present study to prevent selection bias when comparing survival between patients treated with platinum-based and platinum-free chemotherapy. Patients treated with other non-standard regimens (n = 20) were also excluded because only less than five patients were treated by each regimen (Additional file 1: Table S5). Although these non-standard regimens could be further divided into platinum-based or platinum-free regimens, it was unsuitable to include such a heterogeneous population in the analysis.

This study has several limitations. First, patients with a history of other cancers were excluded from this study. Germline mutations in cancer susceptibility genes not only increase the risk of PDAC but also other cancer types. Thus, possible valuable germline mutations that increase the risk of multiple cancers may be lost in our cohort. Second, we only used 80 genes associated with pancreas germline mutations based on the literature review, other oncogenic genes may also contribute to the development of pancreatic cancer. The definition of HR genes was also based on a literature review. The board definition of homologous recombination deficiency (HRD) consists of not only gene mutations but also the identification of genomic scar such as loss of heterozygosity, number of telomeric imbalances, or large-scale transitions which was not included in the current analysis [63]. As a result, some patients with HRD may have been classified as non-HRD in our cohort. While there is currently no consensus on the definition of HRD, the harmonization of an HRD definition will be a critical issue in the future. Third, the family history of patients was self-reported, which may limit the accuracy of the results. Finally, this was an observational study without treatment interventions, and genomic information was not available at the time of treatment onset. Therefore, physicians selected the first-line regimen based on the patient’s condition and preference, potentially introducing bias. This is reflected in a trend favoring the 1L platinum-based group, characterized by factors such as younger age (*P* = 0.091), a higher proportion in stage III than stage IV (*P* = 0.075), lower baseline CA19-9 levels (*P* = 0.072), and greater enrollment in clinical trials (*P* = 0.052), although these differences are not statistically significant. Furthermore, the patient number in the *gHR*^*mut*^ cohort was very limited, comprising only 32 patients. As a result, a prospective validation clinical trial with adequate case number to determine whether *gHR*^*mut*^ could guide the first-line treatment selection in advanced PDAC is warranted.

## Conclusions

Our results identified the germline mutation profiles in Asian populations with pancreatic cancer, such mutations are important not only in the Western population but also in the Asian population. The known and/or unidentified mutations found in our study provide novel insights into the molecular pathogenesis of pancreatic cancer. Our results may help in the prevention, early diagnosis, and risk management of pancreatic cancer. Despite the limited number of cases in the current study, the observed significant survival benefits in patients with *gHR*^*mut*^ treated with platinum-based chemotherapy are consistent with earlier reports. This highlights the importance of germline testing for all patients with advanced PDAC at the time of diagnosis.

### Supplementary Information

**Additional file 1: Figure S1.** Median overall survival of 527 PDAC patients with or without germline gene mutations in different stage. **Figure S2.** Median overall survival of 107 stage III PDAC patients with or without conversion surgery. **Figure S3.** Timeline comparison between the investigator-initiated trials for pancreatic cancer in Taiwan and the corresponding global phase III trials. **Figure S4.** The distribution of the most commonly used regimen by year. An arrow indicates the time of nab-paclitaxel reimbursement in Taiwan. **Figure S5.** Median overall survival of 320 PDAC patients with or without enrollment in clinical trials. **Figure S6.** (A) Median overall survival of 363 stage III/IV PDAC patients treated by different regimens. (B) Median overall survival of 363 stage III/IV PDAC patients with or without HR gene mutations treated with 1L platinum-based or non-platinum-based chemotherapy. **Table S1.** Categories of the 80 candidate cancer-associated genes analyzed for germline mutations in PDAC patients. Genes that have overlapping properties are listed only once and were classified as low-, moderate-, high- and recessive penetrance. **Table S2.** Pathogenic and likely pathogenic germline variants in susceptibility genes. **Table S3.** Family history of cancer in 527 PDAC patients. **Table S4.** Demographics of 32 patients with germline mutation in 12 homologous recombination genes treated with first-line platinum chemotherapy or non-platinum chemotherapy. **Table S5**. List of all regimen used in 368 stage III/IV PDAC patients.

## Data Availability

All data generated or analyzed during this study are available from the corresponding author on reasonable request.

## Abbreviations

ACMG: American College of Medical Genetics and Genomics
MAF: Minor allele frequency
VUS: Variants of uncertain significance
HR: Homologous recombination
IQR: Interquartile range
SLOG: S1, leucovorin, oxaliplatin, and gemcitabine
mFOLFIRINOX: Folinic acid, fluorouracil, irinotecan, and oxaliplatin
GOFL: Gemcitabine, oxaliplatin, fluorouracil, and leucovorin
Gem: Gemcitabine
