# Supplementary material for: Germline mutations of homologous recombination genes and clinical outcomes in pancreatic cancer: a multicenter study in Taiwan
Source: J Biomed Sci. 2024 Feb 13;31:21. doi: 10.1186/s12929-024-01008-7 (PMC10865564; doi:10.1186/s12929-024-01008-7)
Supplement: Supplementary file 1 — Additional file 1: Figure S1. Median overall survival of 527 PDAC patients with or without germline gene mutations in different stage. Figure S2. Median overall survival of 107 stage III PDAC patients with or without conversion surgery. Figure S3. Timeline comparison between the investigator-initiated trials for pancreatic cancer in Taiwan and the corresponding global phase III trials. Figure S4. The distribution of the most commonly used regimen by year. An arrow indicates the time of nab-paclitaxel reimbursement in Taiwan. Figure S5. Median overall survival of 320 PDAC patients with or without enrollment in clinical trials. Figure S6. (A) Median overall survival of 363 stage III/IV PDAC patients treated by different regimens. (B) Median overall survival of 363 stage III/IV PDAC patients with or without HR gene mutations treated with 1L platinum-based or non-platinum-based chemotherapy. Table S1. Categories of the 80 candidate cancer-associated genes analyzed for germline mutations in PDAC patients. Genes that have overlapping properties are listed only once and were classified as low-, moderate-, high- and recessive penetrance. Table S2. Pathogenic and likely pathogenic germline variants in susceptibility genes. Table S3. Family history of cancer in 527 PDAC patients. Table S4. Demographics of 32 patients with germline mutation in 12 homologous recombination genes treated with first-line platinum chemotherapy or non-platinum chemotherapy. Table S5. List of all regimen used in 368 stage III/IV PDAC patients. [file 12929_2024_1008_MOESM1_ESM.zip › 12929_2024_1008_MOESM1_ESM/Additional file 1 Fig. S6.docx]

**A.**


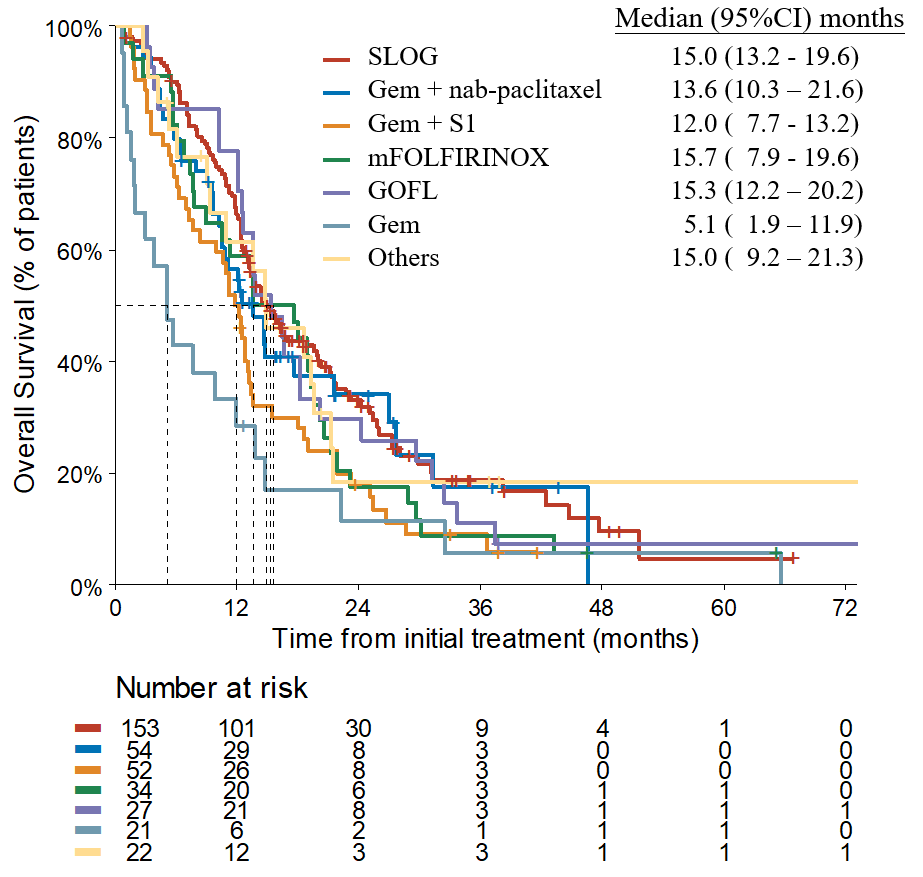


**B.**


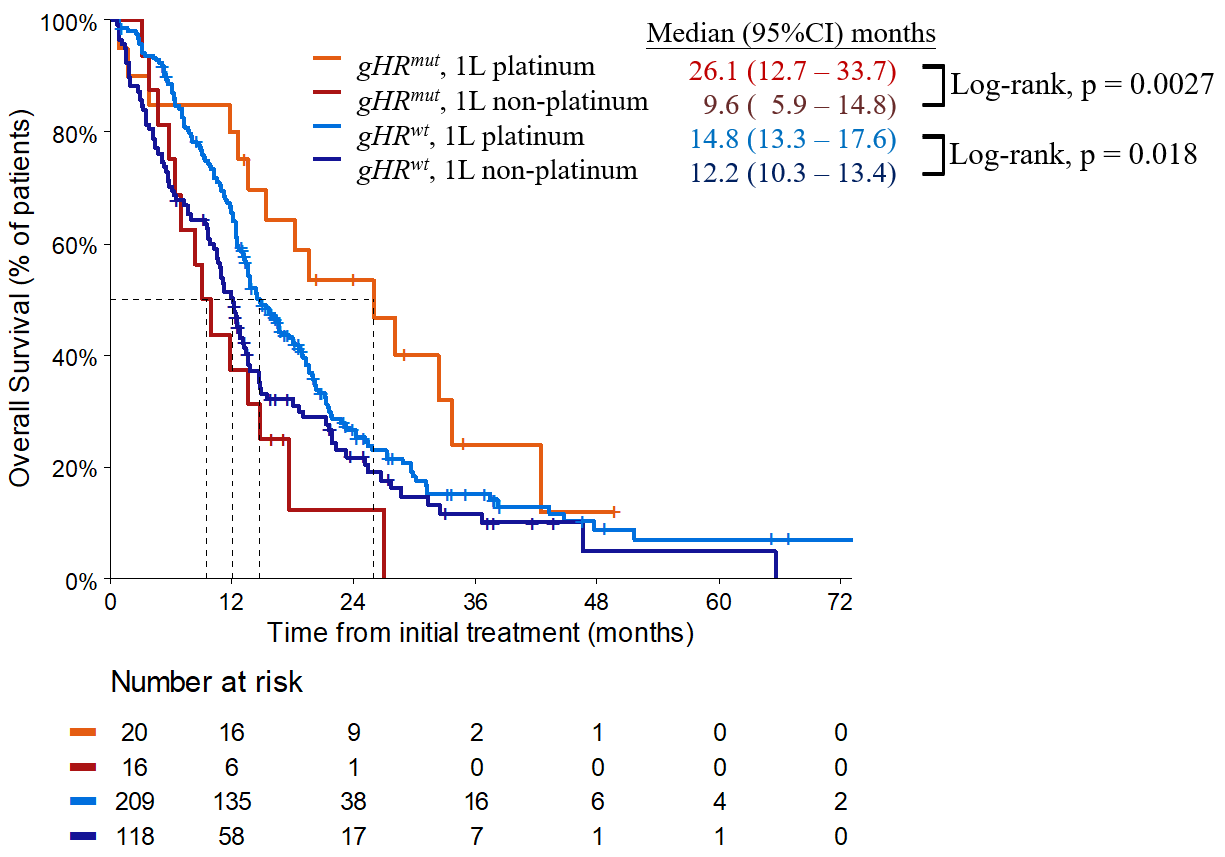


**Figure S6-** (A) Median overall survival of 363 stage III/IV PDAC patients treated by different regimens. (B) Median overall survival of 363 stage III/IV PDAC patients with or without HR gene mutations treated with 1L platinum-based or non-platinum-based chemotherapy.
